# Dr. Walker on Mr. Taunton's Indictment, with the Editor's Remarks

**Published:** 1816-03

**Authors:** 


### *For the London Medical and Physical Journal.*

### Dr. Walker *on Mr. Taunton's Indictment, with the Editor's Remarks.*

YOUR account of the case of "King *versus* Taunton," page 68, was to me extremely satisfactory, inasmuch as it afforded the solution of an important question in medical jurisprudence, viz. how far we are obliged to attempt to prevent the spreading of contagion on occasions where the laws of quarantine do not apply. I was one of the witnesses, all medical, subpœnaed by the defendant as exculpatory evidence, ready to prove that he had been, from the first, a supporter of vaccination, ere some members of the Government Institution were yet converted. (*Par paranthese*, allow me to observe, our summons had been served with a shilling; and we repaired to court under the hope of defeating a conspiracy; while the witnesses on the part of the prosecution received each his guinea, together with his summons.) The plaintiff's witnesses did not appear in court. Had the National Vaccine Establishment again felt themselves drawn into a scrape, now in the Court of King's Bench, as before in the House of Lords (vol. xxxiv. p. 368), by their secretary? and on that account did the Attorney-General, 'on the behalf of his client,' page 68, 'make the most polite retreat in his power?' One reflection I cannot help drawing from the language of the Attorney-General, which had struck me with astonishment in court. A British subject stands accused of a misdemeanor, *quelconquè*—a bill is found against him by a jury of his countrymen—the fact is notorious—the culprit falls under suspicion; yet the plaintiff in the case takes upon him to stop proceedings. He hazards, in the *interim*, the public safety, if the culprit be a criminal; or may injure his reputation if innocent, by withholding from him the opportunity of proving that he is so. Verily, the laws of our country, the best, perhaps, in Europe, ought not thus to be sported with. While a plaintiff, without consent of defendant, can step in between the two juries of his country, and arrest the progress of the law, depend on it there is yet 'something rotten in the state of Denmark.'

*Jan.* 1816.

P.S.—If you really wish to show your impartiality, as you profess in your Journal, now lying before me (Library of the London Institution, 6, ii, 1816), and are much obliged to your correspondent, you will also admit this *coup de plume*, not anonymous; and, perhaps, perhaps, also the information that this is the third time I have been dragged by subpœna into court, from my daily business of vaccination, through this same troublesome agent of mischief, on all of which occasions he had been accuser or instigator of accusation, and on all of which occasions he had been baffled by 'dismissal,' or, as he is so tenacious for *literal* accuracy, by 'the standing' over of the case;' and all the cases have been equally troublesome and vexatious.

---

The powerful calls on our impartiality in the above paper, which may be entitled Walker *v.* Murray, in the matter of the King *v.* Taunton, have induced us to protract the subject thus far; but we trust it will end here. With this view, we subjoin the following report from the Small-Pox Hospital, bearing somewhat on the question, and we hope tending to satisfy all parties.—Edit.

A meeting was held during the current month by the governors of the Small-Pox Hospital, to consider whether the practice of inoculation should be entirely discontinued at that house. The principal argument in favour of the proposition was, that to continue inoculation at such an establishment, even though confined to patients in the house, would be to sanction a practice which ought to be exploded. On the other side it was urged, that according to the opinions of the learned judges and the attorney-general, there is nothing illegal in inoculation if the patient is not exposed to the public, that already the numbers inoculated at the hospital were greatly reduced, and those vaccinated increased in a greater proportion. That whilst the rich can always inoculate and seclude themselves from the public, the poor from their confined dwellings have not the same advantage; that the hospital is a means of lessening that jealousy which the poor might otherwise feel, and from the last report (see our preceding journal) there is every reason to believe that inoculation would fall into disuse the sooner from being left to itself. The governors, therefore, decided to make no alteration in the present practice, and that inoculation should be still continued to in-patients.

